# ECG heartbeat classification using progressive moving average transform

**DOI:** 10.1038/s41598-025-88119-9

**Published:** 2025-02-04

**Authors:** Rabah Mokhtari, Samir Brahim Belhouari, Khelil Kassoul, Abderraouf Hocini

**Affiliations:** 1 Computer Science Department, Faculty of Mathematics and Computer Science, University of M’sila, PO Box 166, Ichbilia, 28000 M’sila, Algeria; 2 Division of Information and Computing Technology, College of Science and Engineering, Hamad Ben Khalifa University, Doha, Qatar; 3 Geneva School of Business Administration, University of Applied Sciences Western Switzerland HES-SO, 1227 Geneva, Switzerland; 4 Computer Science Department, University of M’sila, M’sila, Algeria

**Keywords:** Computer science, Machine learning

## Abstract

This paper presents the Progressive Moving Average Transform (PMAT), a novel signal transformation method for converting time-domain signals into 2D representations by progressively computing Moving Averages (MAs) with varying window sizes. The approach aims to enhance signal analysis and classification, particularly in the context of heartbeat classification. Our approach integrates PMAT with a 2D-Convolutional Neural Network (CNN) model for the classification of ECG heartbeat signals. The 2D-CNN model is employed to extract meaningful features from the transformed 2D representations and classify them efficiently. To assess the effectiveness of our approach, we conducted extensive simulations utilizing three widely-used databases: the MIT-BIH database and the INCART database, chosen to cover a wide range of heartbeats. Our experiments involved classifying more than 6 heartbeat types grouped into three main classes. Results indicate high accuracy and F1-scores, with 99.09% accuracy and 92.13% F1-score for MIT-BIH, and 98.37% accuracy and 79.37% F1-score for INCART. Notably, the method demonstrates robustness when trained on one database and tested on another, achieving accuracy rates exceeding 95% in both cases. Specifically, the method achieves 96% accuracy when trained on MIT-BIH and tested on the ST-T European database. These findings underscore the effectiveness and stability of the proposed approach in accurately classifying heartbeats across different datasets, suggesting its potential for practical implementation in medical diagnostics and healthcare systems.

## Introduction

The heart plays a crucial role in sustaining human life by circulating oxygen and nutrient-rich blood throughout the body. Any dysfunction in its operation can lead to severe consequences, potentially fatal. According to the World Health Organization (WHO), cardiovascular diseases are the leading cause of death globally, claiming over 17 million lives annually. Preventing heart-related complications remains a paramount healthcare objective.

The electrocardiogram (ECG) remains a cornerstone in diagnosing heart irregularities, even more than a century after its inception by Willem Einthoven^1^. Einthoven’s pioneering work identified distinctive patterns in heart rhythms, forming the basis of ECG analysis. Comprising a sequence of waves - the P wave, QRS complex, and T wave - the ECG provides vital insights into heart health through the size, shape, and duration of these waves.

In recent years, Convolutional Neural Networks (CNNs) have emerged as potent tools for image classification, leveraging their capacity to discern intricate features from input images. Applied in the domain of cardiovascular disease (CVD) classification, various transformations are employed to convert ECG signals from the time domain to a two-dimensional representation. Notably, the Continuous Wavelet Transform (CWT)^2^ and Short-Time Fourier Transform (STFT)^3^ are commonly utilized for this purpose.

In this study, we introduce a novel transformation termed the Progressive Moving Average Transform (PMAT). This algorithm facilitates the conversion of discrete ECG signals into a 2D format by iteratively computing the Moving Average (MA) across different window sizes. The resultant representation features time along one dimension and the utilized window sizes along the other. Moreover, we present a practical application of the PMAT transform in classifying ECG heartbeat signals. Our methodology entails transforming ECG data into 2D images using PMAT, followed by feature extraction and classification using a 2D-CNN model. We conducted five experiments across three distinct databases - the MIT-BIH^4^, INCART^5^, and European ST-T^6^ databases, each containing similar heartbeat types. We study more than 6 heartbeat types grouped into three main classes Non-ectopic beats (N), Supraventricular ectopic beats (S), and Ventricular ectopic beats (V) following the guidelines provided by the recommendation of the Association for Advanced Medical Instrumentation (AAMI)^7^. These experiments included training and testing our method separately on each database, as well as alternating between databases for training and testing. The most challenging experiments conducted in this study involved training our solution on either the MIT-BIH or INCART database and subsequently testing it on the other (or on European ST-T).

## Related works

Electrocardiogram (ECG) classification has been extensively studied, with various methods proposed to improve accuracy and robustness. Early works, such as the works of Chazal et al.^8^ and Zhao and Zhang^9^. Chazal et al.^8^ utilized ECG morphology and heartbeat interval features, applying linear discriminant analysis to classify heartbeats into five ANSI/AAMI classes. This approach compared twelve configurations of feature sets derived from two ECG leads to optimize a statistical classifier model. However, this method was tested exclusively on the MIT-BIH database and demonstrated limited accuracy, raising concerns about its generalizability. Zhao and Zhang^9^ proposed a hybrid feature extraction approach for ECG data, combining two methods. The first method employed wavelet transform to extract coefficients as features from each ECG segment, capturing frequency-domain characteristics. The second method applied autoregressive modeling to capture the temporal structures of ECG waveforms. These features were subsequently used to train and validate an SVM classifier. However, the method was tested on a very limited dataset, extracted from a small number of records from the MIT-BIH database, which raises concerns about its generalizability to broader datasets.

As works utilizing methods similar to our proposed PMAT transform, we highlight two relevant studies: The first study, proposed by Huang et al.^3^, employed the Short-Time Fourier Transform (STFT) to convert ECG heartbeats into two-dimensional scalograms. A Convolutional Neural Network (CNN) with three convolutional layers was used to extract features from these scalograms and classify five types of heartbeats from the MIT-BIH database. To ensure optimal performance, the authors conducted a series of experiments to optimize the learning rate and batch size. However, this method was tested on a very limited subset, which raises concerns about its generalizability. The second study, proposed by Yoon et al.^10^, developed a bimodal CNN for cardiovascular disease classification by co-training grayscale images and scalograms of ECG data. The scalograms were generated using the Continuous Wavelet Transform (CWT) and subsequently converted into grayscale images. The CNN model utilized these dual inputs to classify four classes regrouping 11 ECG rhythms from a 12-lead ECG database. The model achieved promising results, demonstrating significant diagnostic potential for cardiovascular diseases, particularly given the size of the database. However, it still requires testing on other external databases to ensure its robustness and generalizability.

## Preliminaries

This section outlines several techniques employed within the scope of this paper.

### Moving average

The concept of Moving Average (MA) was initially introduced in 1965 and has since found widespread application across diverse domains for analyzing time series data^11^. For instance, in trading, a common practice involves employing two moving averages - a shorter-term moving average and a longer-term moving average - in tandem to identify trading events. In the realm of electrocardiography (ECG), recent research has demonstrated the efficacy of MA in accurately pinpointing desired peaks within ECG signals^12^. The moving average is an effective tool for capturing temporal smoothing, which is a crucial feature in ECG signal analysis. We believe that using multiple windows, as described in the expression (1), to visualize the signal in a two-dimensional space provides a new perspective on ECG data analysis. This approach allows for the application of powerful techniques like CNNs for classification purposes.

The MA of a discrete signal *X* of length $n>1$ is defined as follow:

1 $$\begin{aligned} MA(X,j,w) = \frac{\sum _{i=j}^{j+w-1}(X_i)}{w} \end{aligned}$$

where, the index $j$ ranges from 1 to $n-1$, representing consecutive moving averages. The window size $w$ ranges from 2 to $n$, denoting the size of the moving average window. $X_i$ signifies the $i^{th}$ consecutive sample of $X$. The total number of MAs equals $n - w + 1$. These computed moving averages, denoted as $MA_1, MA_2, \ldots , MA_{n-w+1}$, collectively form the output signal $Y$.

#### Example

Given a discrete signal *X* of six values where, $X=(6 \quad 4 \quad 2 \quad 0 \quad 2 \quad 12)$. Using a window size $w=2$, we can calculate five consecutive moving averages. The five values are given as follow:

- $$MA(X,1,2) = \frac{X_1+X_2}{2} =5$$,
- $$MA(X,2,2) = \frac{X_2+X_3}{2} =3$$,
- $$MA(X,3,2) = \frac{X_3+X_4}{2} =1$$,
- $$MA(X,4,2) = \frac{X_4+X_5}{2} =1$$, and
- $$MA(X,5,2) = \frac{X_5+X_6}{2} =7$$

forming the output signal $Y1 = (5 \quad 3 \quad 1 \quad 1 \quad 7)$.

Similarly, when $w = 3$, the resulting signal is: $Y2=(\frac{X_1+X_2+X_3}{3} \quad \frac{X_2+X_3+X_4}{3} \quad \frac{X_3+X_4+X_5}{3} \quad \frac{X_4+X_5+X_6}{3}) = (4 \quad 2 \quad \nicefrac {4}{3} \quad \nicefrac {14}{3})$

The lengths of *Y*1 and *Y*2 are 5 and 4 respectively. Importantly, it’s worth noting that the length of the output signal, which is determined by $n - w + 1$, consistently remains shorter than the length of the original signal *X*.

### Convolution neural networks

Convolutional Neural Networks (CNNs) represent a class of neural networks extensively utilized for tasks such as image classification, object detection, and various computer vision applications. A distinguishing characteristic of CNNs lies in their capability to autonomously extract pertinent features from raw input data, particularly images. The inception of CNNs can be attributed to Yann LeCun et al.^13^, who introduced the concept, demonstrating its prowess in achieving high accuracy in digit handwriting recognition. The efficacy displayed by CNNs in document recognition spurred further exploration, culminating in the evolution of contemporary deep learning models. Notably, the seminal success of AlexNet^14^ in employing deep CNNs for image classification significantly popularized their adoption in this field.

CNNs possess the inherent ability to automatically derive discriminative features during the training phase, rendering them particularly well-suited for classification endeavors. The effectiveness of CNNs in image and video analysis hinges on several pivotal concepts: sparse interaction, parameter sharing, pooling, and the multilayer structure^15^. These elements collectively contribute to CNNs’ proficiency in processing and extracting meaningful information from visual data.

### Electrocardiogram (ECG)

**Figure 1:**
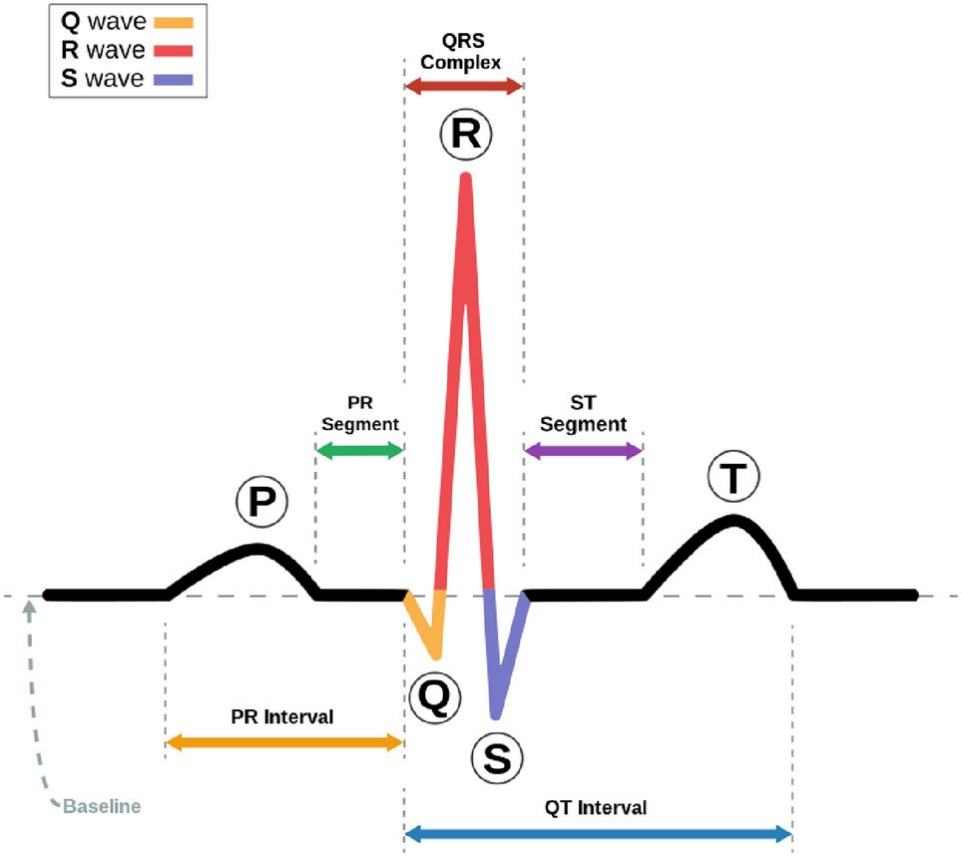
Heartbeat and QRS complex.

Electrocardiogram (ECG) recordings capture a series of heartbeats, each characterized by distinct waves: P, Q, R, S, and T waves (refer to Fig. 1). The P wave corresponds to atrial depolarization, while the QRS complex, comprising the Q, R, and S waves, reflects ventricular depolarization. Lastly, the T wave signifies ventricular depolarization. The R-peak denotes the highest positive deflection on the R wave within the QRS complex. Due to its prominent amplitude, it is the most easily identifiable feature^16^. In this paper, we utilize the RR interval, defined as the duration between two successive R peaks. The RR interval serves as a fundamental feature extensively employed in ECG signal classification studies^17^.

## Progressive moving average transform

This section presents our innovative transform, the Progressive Moving Average Transform (PMAT), which is tailored for converting discrete signals from their original time domain representation (1D) into a 2D format. The PMAT algorithm achieves this by progressively computing the MA of the input signal using varying window sizes. Through the application of the PMAT algorithm, the signal undergoes a transformation from its initial 1D time domain representation to a 2D representation. Within this transformed representation, the first dimension retains the temporal characteristics of the signal, corresponding to time. Meanwhile, the second dimension signifies the window sizes utilized by the PMAT transform, providing additional context to the transformed data. The computational complexity of the PMAT algorithm is $O(N^2)$, where N is the length of the input signal.

To seamlessly integrate the MA computation into our PMAT transform, we develop a padding technique. This technique ensures uniform output signal lengths, facilitating consistent application of the transformation across different input signals.

### Left, right, and centered padded moving averages

In the context of the MA definition, it is essential to note that the length of the resulting output signal $Y$ is determined by the expression $n - w + 1$, where $n$ represents the length of the original signal $X$, and $w$ corresponds to the window size. It’s important to highlight that the length of this output signal consistently remains shorter than that of $X$. To ensure equal signal lengths, a practical approach involves padding either the left or right side of the new signal $Y$ with $w-1$ instances of its initial or final value, respectively. This technique guarantees compatibility in signal lengths. Referring to Formula 1, it’s evident that the initial and final moving averages correspond to indices $j=1$ and $j=n-w+1$, respectively.

Let $LPMA(X,k,w)$ denote the Left Padded MA, $RPMA(X,k,w)$ denote the Right Padded MA, and $CPMA(X,k,w)$ represent the Centered Padded MA. These are defined as follows:

2 $$\begin{aligned} LPMA(X,k,w) = {\left\{ \begin{array}{ll} MA(X,1,w) & \hbox { if}\ k \le w \\ MA(X,k-w+1,w) & \text {otherwise} \end{array}\right. } \end{aligned}$$

3 $$\begin{aligned} RPMA(X,k,w) = {\left\{ \begin{array}{ll} MA(X,n-w+1, w) & \hbox { if}\ k > n-w \\ MA(X,k,w) & \text {otherwise} \end{array}\right. } \end{aligned}$$

4 $$\begin{aligned} CPMA(X,k,w) = {\left\{ \begin{array}{ll} MA(X,1, w) & \hbox { if}\ k \le \left\lfloor \frac{w-1}{2} \right\rfloor + p \\ MA(X,n-w+1, w) & \hbox { if}\ k > n-\left\lfloor \frac{w-1}{2} \right\rfloor \\ MA(X,k-\left\lfloor \frac{w-1}{2} \right\rfloor - p,w) & \text {otherwise} \end{array}\right. } \end{aligned}$$

where, $1 \le k \le n$ and $2 \le w \le n$; $p$ equals 1 if $w$ is even, otherwise $p$ is 0; and $\left\lfloor \frac{w}{2} \right\rfloor$ denotes the result of integer division of $w$ by 2.

#### Example

Given the discrete signal *X* consisting of six values, as illustrated earlier:

$X=(6 \quad 4 \quad 2 \quad 0 \quad 2 \quad 12)$. For *LPMA* of *X* with window sizes $w=2$ and $w=3$, we obtain the signals:

*LPMA* with $w=2$: $({5} \quad 5 \quad 3 \quad 1 \quad 1 \quad 7)$, *LPMA* with $w=3$: $({4} \quad {4} \quad 4 \quad 2 \quad \nicefrac {4}{3} \quad \nicefrac {14}{3})$. Similarly, for *RPMA* and *CPMA* of *X* with the same window sizes, the resulting signals are:

*RPMA* with $w=2$: $(5 \quad 3 \quad 1 \quad 1 \quad 7 \quad {7})$, *RPMA* with $w=3$: $(4 \quad 2 \quad \nicefrac {4}{3} \quad \nicefrac {14}{3} \quad {\nicefrac {14}{3}} \quad {\nicefrac {14}{3}})$.

*CPMA* with $w=2$: $({5} \quad 5 \quad 3 \quad 1 \quad 1 \quad 7)$, *CPMA* with $w=3$: $({4} \quad 4 \quad 2 \quad \nicefrac {4}{3} \quad \nicefrac {14}{3} \quad {\nicefrac {14}{3}})$.

In each case, the red values indicate the padded values introduced by the padding techniques.

Derived from the Formula 2, 3, and 4 the Progressive Moving Average Transform (PMAT) encompasses three distinct variants: Left PMAT (LPMAT), Right PMAT (RPMAT), and Centered PMAT (CPMAT).

### Left PMAT

Given a discrete signal *X* with a length of $n>1$, where $X_i$ represents the $i_{th}$ consecutive sample. Left PMAT (LPMAT) utilizes the Left Padded Moving Average (LPMA) defined in Formula 2, and its definition is presented as follows:

5 $$\begin{aligned} LPMAT(X,i,w) = {\left\{ \begin{array}{ll} X_i & \hbox { if}\ w=1 \\ LPMA(X,i,w) & \text {otherwise} \end{array}\right. } \end{aligned}$$

where $1 \le i \le n$, and $1 \le w \le w_{max}$. Here, $w_{max} \le n$ represents a fixed maximum window size that *w* can attain.

The outcome of the Left PMAT transformation manifests as a two-dimensional representation, incorporating discrete time points and window sizes. The ensuing matrix represents the transformation of the provided discrete signal $X=(6 \quad 4 \quad 2 \quad 0 \quad 2 \quad 12)$ using the maximum window size of $w_{\text {max}} = 4$.

$$\begin{bmatrix} {3} & {3} & {3} & 3 & 2 & 4 \\ {4} & {4} & 4 & 2 & \nicefrac {4}{3} & \nicefrac {14}{3} \\ {5} & 5 & 3 & 1 & 1 & 7 \\ 6 & 4 & 2 & 0 & 2 & 12 \\ \end{bmatrix} \begin{array}{c} \text {w=4}\\ \text {w=3}\\ \text {w=2}\\ \text {w=1} \end{array}$$

The initial row of this matrix contains the signal *X* as is, while the subsequent rows encompass the Left Padded Moving Averages for various window sizes, ranging from $2 \le w \le w_{max} = 4$.

### Right PMAT

Similarly to the LPMAT, the Right PMAT (RPMAT) transform utilizes the Right Padded Moving Average (RPMA) defined in Formula 3. The definition of RPMAT is outlined as follows:

6 $$\begin{aligned} RPMAT(X,i,w) = {\left\{ \begin{array}{ll} X_i & \text {if w=1} \\ RPMA(X,i,w) & \text {otherwise} \end{array}\right. } \end{aligned}$$

where *X* is a discrete signal with a length of $n>1$, and $X_i$ represents the $i^{th}$ consecutive sample. The range of indices is $1 \le i \le n$, and for the window size, $1 \le w \le w_{max}$, where $w_{max} \le n$ denotes a predetermined maximum window size that *w* can achieve.

The outcome of the Right PMAT transformation manifests as a two-dimensional representation, incorporating discrete time points and window sizes. The ensuing matrix represents the transformation of the provided discrete signal $X=(6 \quad 4 \quad 2 \quad 0 \quad 2 \quad 12)$ using the maximum window size of $w_{\text {max}} = 4$.

$$\begin{bmatrix} 3 & 2 & 4 & {4} & {4} & {4} \\ 3 & 2 & \nicefrac {4}{3} & \nicefrac {14}{3} & {\nicefrac {14}{3}} & {\nicefrac {14}{3}} \\ 5 & 3 & 1 & 1 & 7 & {7} \\ 6 & 4 & 2 & 0 & 2 & 12 \\ \end{bmatrix} \begin{array}{c} {w=4}\\ {w=3}\\ {w=2}\\ {w=1} \end{array}$$

### Centered PMAT

Based on the two variants of the CPMA procedure defined in Formula 4, Centered PMAT (CPMAT) of a discrete signal *X*, with a length of $n>1$, is introduced and defined as follows:

7 $$\begin{aligned} CPMAT(X,i,w) = {\left\{ \begin{array}{ll} X_i & \text {if } w=1 \\ CPMA(X,i,w) & \text {otherwise} \end{array}\right. } \end{aligned}$$

Where $X_i$ represents the $i_{th}$ consecutive sample, $1 \le i \le n$, and $1 \le w \le w_{max}$. Here, $w_{max} \le n$ represents a fixed maximum window size that *w* can attain.

Considering for example the discrete signal $X=(6 \quad 4 \quad 2 \quad 0 \quad 2 \quad 12)$ employed earlier, along with the resulting outputs given by CPMA for $w \le 4$, the CPMAT of *X* can be described as follows:

$$\begin{bmatrix} {3} & {3} & 3 & 2 & 4 & {4} \\ {4} & 4 & 2 & \nicefrac {4}{3} & \nicefrac {14}{3} & {\nicefrac {14}{3}} \\ {5} & 5 & 3 & 1 & 1 & 7 \\ 6 & 4 & 2 & 0 & 2 & 12 \\ \end{bmatrix} \begin{array}{c} \text {w=4}\\ \text {w=3}\\ \text {w=2}\\ \text {w=1} \end{array}$$

### Illustrations of ECG heartbeat transformation

**Figure 2:**
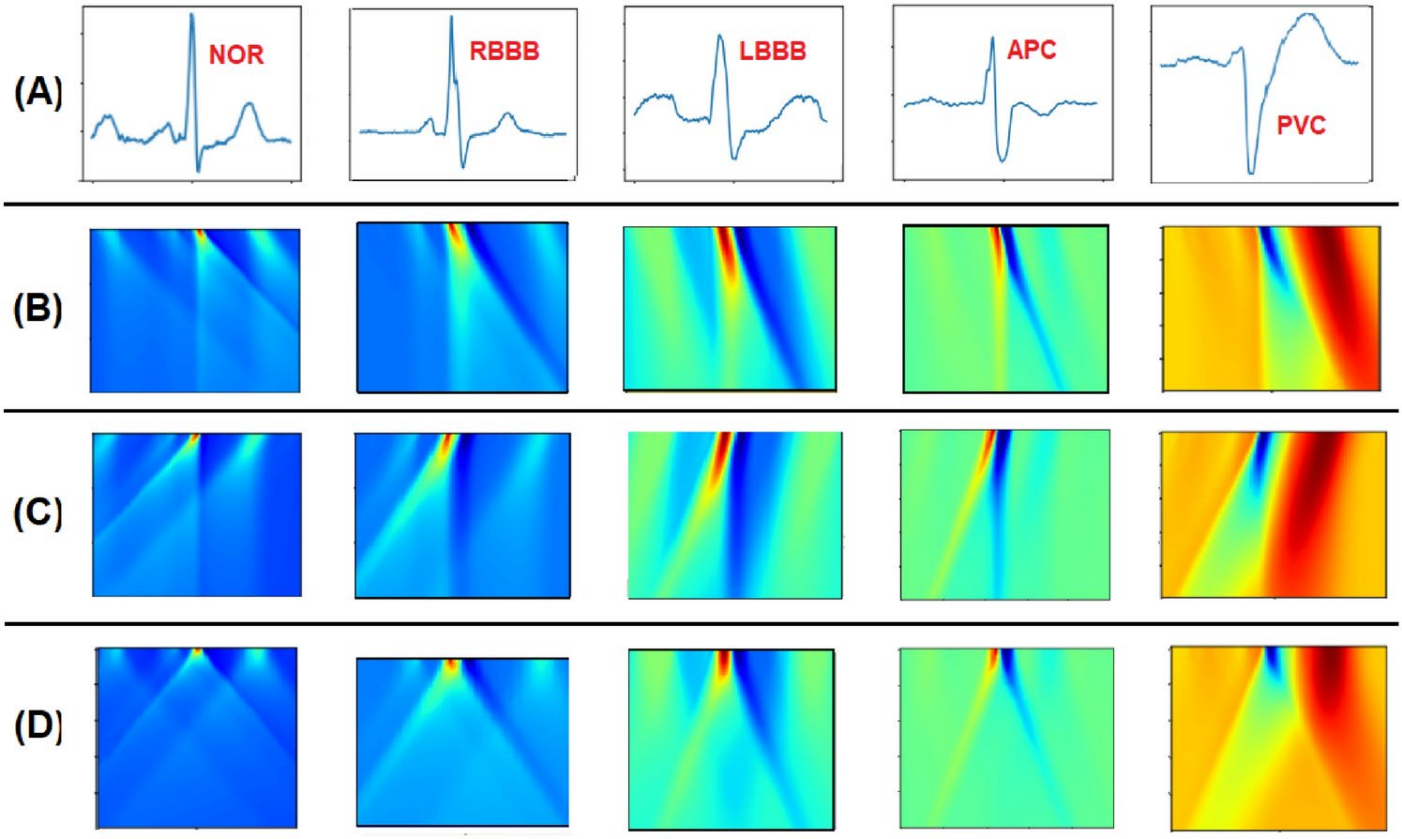
Transformation of ECG heartbeat signals using PMAT transform (**A**) represents a set of normal and abnormal heartbeats, (**B**) The transformed heartbeat signals using LPMAT, (**C**) The transformed heartbeat signals using RPMAT, and (**D**) The transformed heartbeat signals using CPMAT.

This section illustrates examples of the application of the three PMAT transform variants to ECG heartbeats. Figure 2 comprises four rows. The first row **(A)** showcases five distinct heartbeat types: Normal (NOR), Left Bundle Branch Block (LBBB), Right Bundle Branch Block (RBBB), Atrial Premature Contraction (APC), and Premature Ventricular Contraction (PVC). Subsequent rows **(B)**, **(C)**, and **(D)** depict images of the transformed heartbeats. Row **(B)** illustrates the transformation using LPMAT, row **(C)** using RPMAT, and row **(D)** using CPMAT.

To facilitate a visual comparison between PMAT and other transforms, we introduce two similar techniques: Continuous Wavelet Transform (CWT) and Short-Time Fourier Transform (STFT). CWT employs a wavelet, which is a small wave-like function, to generate a time-scale scalogram from a signal. The wavelet is systematically scaled and shifted across the signal over time to capture information at various times and scales. On the other hand, STFT offers insights into the temporal localization of frequency components in situations where the frequency components of a signal vary over time. STFT segments the signal into short and time-overlapped segments, subsequently computing the Fourier Transform of each segment. This process effectively generates a visual representation of the signal, illustrating how frequency components evolve over time.

**Figure 3:**
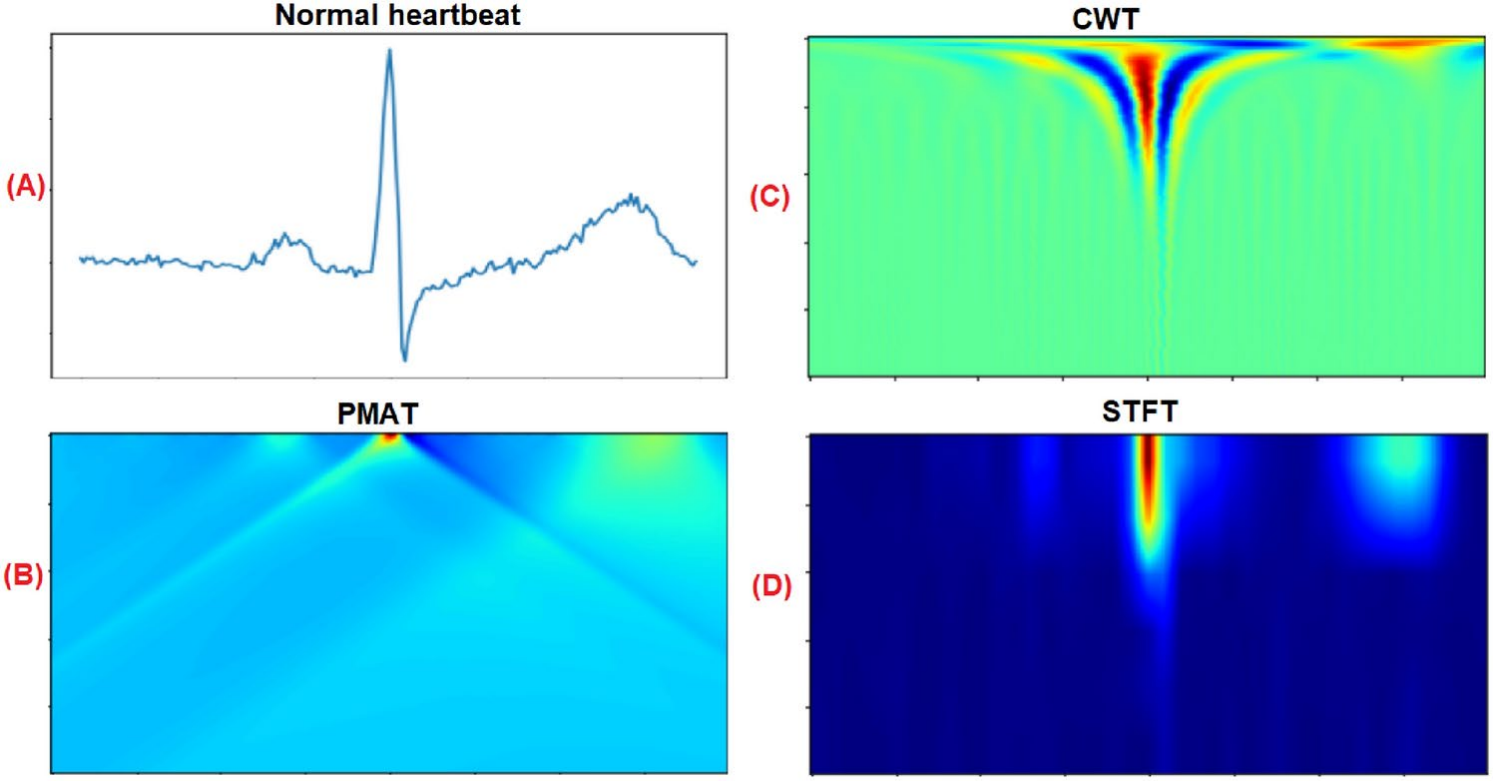
Transformation of a Normal heartbeat signals using PMAT, CWT, and STFT transforms.

Figure 3 presents an example of the transformation of a normal heartbeat using PMAT, CWT, and STFT. By examining the three output images corresponding to these techniques, one can easily discern the temporal localization of crucial information such as the P, R, and T waves. The distinction between the shapes of the P and T waves becomes evident, particularly when comparing the images generated by PMAT and STFT.

## Classification of heartbeats using PMAT and 2D-CNN model

This section introduces an approach that utilizes PMAT alongside a 2D-CNN model for heartbeat classification. To demonstrate the effectiveness of our novel transform, we utilize three distinct databases. The first is the renowned MIT-BIH database^4^, while the second is the INCART database^18^, and the European ST-T database^6^. Subsequently, the following section delves into the results and offers comparisons with other selected works to validate the efficacy of our approach.

**Figure 4:**
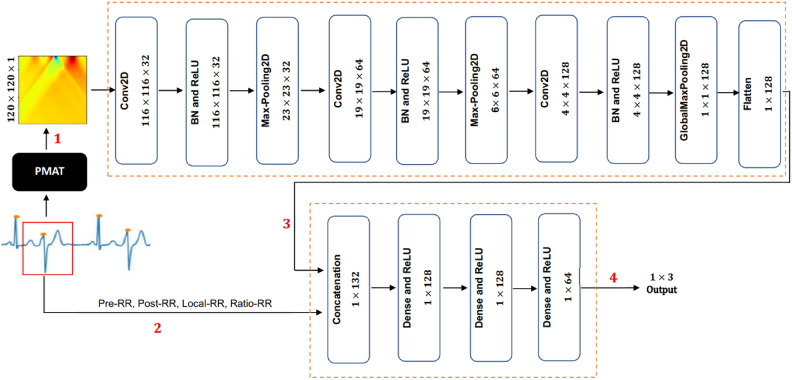
Overview of the approach.

Figure 4 offers an overview of our methodology. For each database, we initiate by extracting heartbeats from denoised ECG signals, leveraging annotated r-peaks. The numbered steps (1 to 5) outline the process our method undergoes:

- Step 1: Employ LPMAT to transform ECG heartbeats into 2D representations (images) by resizing the images to $120 \times 120$.
- Step 2: Extract four handcrafted features Pre-RR, Post-RR, Local-RR, and Ratio-RR. This features are used in^17^. Pre-RR refers to the RR interval between the current heartbeat and the preceding heartbeat. Similarly, the Post-RR represents the RR interval between the current heartbeat and the subsequent heartbeat. Ratio-RR denotes the ratio of the Pre-RR to Post-RR. Furthermore, Local-RR is calculated by computing the average of the ten preceding RR intervals of the current heartbeat. All these four features are measured in second unit.
- Step 3: Utilize sequential 2D-CNN layers to extract 128 features from the transformed heartbeats.
- Step 4: Utilize sequential four dense layers to classify the heartbeats. The first dense layer takes as input the extracted 4 handcrafted features obtained in Step 2 concatenated with the 128 extracted features obtained in Step 3.

### Feature extraction

Step 3 of our methodology, depicted in Fig. 4, employs a 2D-CNN model to extract features from the image generated through PMAT transformation. The architecture of the CNN model comprises multiple layers, including 2D convolutional layers, 2D batch normalization layers, ReLU activation units, 2D max pooling layers, global max pooling layers, and a final flatten layer. These layers collaborate to process the input 2D representation and extract relevant features. The extracted features are then combined with other statistical features, forming the input for the ANN classifier to make predictions.

Our model adopted the same architecture used by Huang et al.^3^. The main difference between our approach and theirs lies in the introduction of the handcrafted features and in the size of the input images. Although their model uses spectrograms with dimensions of 256$\times$256, we utilized PMAT images with dimensions of 120$\times$120. Despite this difference, as described in the results section, our method achieved superior performance.

The first 2D convolutional layer accepts the transformed heartbeat as input, which has dimensions of $120 \times 120$. Utilizing 2D convolutional layers enables the model to learn spatial relationships between adjacent pixels in the 2D representation. Batch normalization layers are employed to normalize the inputs and enhance the stability and efficiency of the training process. ReLU activation functions introduce non-linearity into the model, crucial for learning complex patterns in the data. The 2D max pooling layer reduces the spatial dimensions of the feature maps, while the global max pooling layer aggregates the most salient features across all feature maps.

Subsequently, the flatten layer transforms the output of the previous layers into a one-dimensional feature vector of length 128. These 128 features extracted from each heartbeat are concatenated with four additional features: Pre-RR, Post-RR, Local-RR, and Ratio-RR, all belonging to the same heartbeat. This concatenation of features is then utilized for heartbeat classification. The parameters of these layers and their relationships are detailed in Table 1.

**Table 1:** 2D-CNN model layers and parameters.

No.	Layer name	Kernal size	Filter	Padding	Stride	Output shape
1	$\hbox {Input}^a$	–	–	–	–	$120\times 120\times 1$
2	Conv2D	$5\times 5$	32	0	1	$116\times 116\times 32$
3	BatchNorm2d	–	–	–	–	$116\times 116\times 32$
4	ReLU	–	–	–	–	$116\times 116\times 32$
5	MaxPool2D	$5\times 5$	–	–	5	$23\times 23\times 32$
6	Conv2D	$5\times 5$	64	0	1	$19\times 19\times 64$
7	BatchNorm2d	–	–	–	–	$19\times 19\times 64$
8	ReLU	–	–	–	–	$19\times 19\times 64$
9	MaxPool2D	$3\times 3$	–	–	3	$6\times 6\times 64$
10	Conv2D	$3\times 3$	128	0	1	$4\times 4\times 128$
11	BatchNorm2d	–	–	–	–	$4\times 4\times 128$
12	ReLU	–	–	–	–	$4\times 4\times 128$
13	AMaxPool2$\hbox {d}^b$	–	–	–	–	$1\times 1\times 128$
14	Flatten	–	–	–	–	$1\times 128$
15	Flatten	–	–	–	–	$1\times 128$
16	Flatten	–	–	–	–	$1\times 64$
17	Flatten	–	–	–	–	$1\times 3$

$^{\text {a}}$The input refers to the PMAT transformed heartbeat of size $120\times 120$, $^{\text {b}}$ AMaxPool2d is the Adaptive Max Pooling layer

### Data description and preprocessing

In this study, we perform experiments utilizing three distinct databases: the MIT-BIH arrhythmia database^4^ and the INCART arrhythmia database^5^, and the European ST-T database^6^. The MIT-BIH database is widely acknowledged for evaluating ECG classification methods. ECG signals in the MIT-BIH database are recorded using two leads: modified limb lead II (MLII) and modified lead V1 (occasionally V2 or V5, and in one instance V4)^18^. Conversely, the ECG signals in the INCART database are recorded using the 12 standard leads. The MIT-BIH database comprises 48 half-hour annotated ECG records, obtained from 47 subjects (22 females aged 23–89 years and 25 males aged 32–89 years). Each record is sampled at 360 Hz.

In comparison, the INCART database is larger than the MIT-BIH database. It consists of 75 annotated recordings extracted from 32 Holter records. Each record is 30 minutes long and sampled at 275 Hz^5^. Both databases share almost identical types of heartbeats, and this study focuses solely on the ECGs obtained from the MLII lead.

The European ST-T database annotated 79 records sampled at 250 Hz, each lasting two hours. However, in this study, we only used 47 records from the database, as these are the ones recorded using the MLII lead. This ensures consistency with the lead configurations used in the MIT-BIH database.

#### Filtering and scaling of ECG signals

Clinically obtained ECG signals often suffer from various sources of noise, including baseline wandering, electromyographic interference, and power line interference, which can obscure meaningful information within the raw ECG data^17^.

Baseline wandering, characterized by low-frequency noise in the ECG, is primarily caused by respiration, patient movement, or environmental electrical interference. This noise can significantly hinder the extraction of useful information from the raw ECG signal^19,20^. Therefore, it is imperative to preprocess the signal by filtering out baseline wandering. However, excessive filtering may result in the loss of valuable data. Hence, in this study, we focus solely on removing baseline wandering, as it has a significant impact on ECG classification^20^.

To mitigate the effects of baseline wandering without sacrificing valuable information, we employ two median filters: a 200 ms width median filter and a 600 ms width median filter, as utilized in prior work^17^. Following the filtering process, we scale the amplitude of all consecutive signals $X_j$ using the scaling formula outlined in Eq. (8).

**Figure 5:**
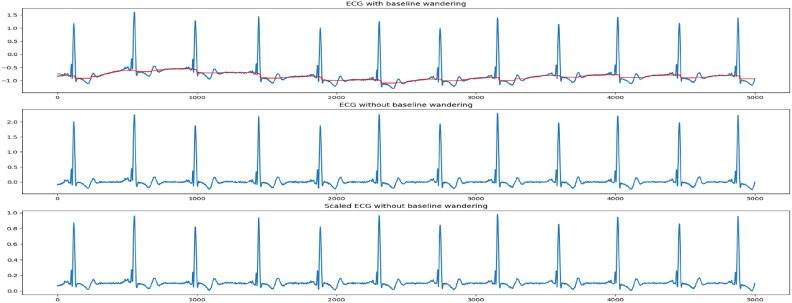
Baseline wandering removal using median filters and scaling of ECG signal from record 124 of the MIT-BIH database. The sub-figure (**A**) represents the baseline of the red color and the raw signal, (**B**) The filtered signal, and (**C**) The scaled signal.

Figure 5 illustrates an example of baseline wandering removal and amplitude scaling of a fragment from record 124 of the MIT-BIH database.

8 $$\begin{aligned} \bar{X_j}(i)= \frac{X_j(i) - min(X_j(i))}{max(X_j) - min(X_j)} \end{aligned}$$

where *j* - index of consecutive signals, *i* - index of consecutive signal samples, $min(X_j)$ - minimum signal amplitude of the filtered signal $X_j$, $max(X_j)$ - maximum signal amplitude of the the filtered signal $X_j$.

Scaling the signal can normalize the amplitude, making it easier to identify and distinguish features of interest, such as the QRS complex waves. The amplitude of any scaled ECG signal $\bar{X}_j$ is between 0 and 1.

## Results discussion and evaluation

### Evaluation metrics

We evaluate the proposed method in terms of three key metrics: accuracy, precision (or positive predictive value PPV), sensitivity (or recall), and F1-score.

Accuracy measures the proportion of correctly classified instances out of the total number of instances. Precision (PPV) quantifies the proportion of true positive predictions among all positive predictions. Sensitivity (Recall) represents the proportion of true positive predictions among all actual positive instances. *F*1-score is the harmonic mean of precision and recall, providing a balanced assessment of the model’s performance.

Accuracy, Precision, Sensitivity, Specificity, and F1-score are determined using the corresponding formulas represented by Eqs. (9), (10), (11), (12), and (13), respectively.

9 $$\begin{aligned} Accuracy = \frac{TP+TN}{TP+TN+FP+FN} \end{aligned}$$

where *TP* and *TN* denote respectively True Positive and True Negative, whereas *FP* and *FN* denote false positive and false negative.

10 $$\begin{aligned} Precision = \frac{TP}{TP + FP} \end{aligned}$$

11 $$\begin{aligned} Sensitivity = \frac{TP}{TP + FN} \end{aligned}$$

12 $$\begin{aligned} Specificity = \frac{TN}{TN + FP} \end{aligned}$$

13 $$\begin{aligned} F1 = 2 \times \frac{Precision \times Sensitivity}{Precision+Sensitivity} \end{aligned}$$

### Dataset splitting approaches

To validate our method and underscore the effectiveness of the PMAT transform, we conduct four experiments. In the first two experiments, we partition the records of different patients within each database into two separate subsets. This partitioning ensures that heartbeats from the same patient are not present in both the training and testing subsets. In the final two experiments, we train the method on one database (either MIT-BIH or INCART) and subsequently test it on the other. Additionally, to evaluate the robustness of our method, we utilize 47 records from the European ST-T database exclusively for testing purposes. These 47 records were selected out of the 79 total records because they were recorded using the MLII lead, ensuring consistency with the leads used in the other databases. This approach offers a robust and more realistic validation of the proposed method.

The remainder of this paper presents a comprehensive evaluation of our method, incorporating all these experimental approaches.

### Classification of heartbeat types

MIT-BIH, INCART, and European ST-T databases use annotations defined by the Association for Advanced Medical Instrumentation (AAMI) to categorize heartbeats into different types^7^. Specifically, the MIT-BIH database annotates 15 types of heartbeat, while the INCART database annotates 11 types, and the European ST-T database annotates 7 types. According to the AAMI guidelines, all types of heartbeat are classified into five main classes. Table 2 illustrates these five classes along with the distribution of heartbeat types in both databases. Notably, the MIT-BIH database exhibits a wider range of heartbeat types compared to the INCART and European ST-T databases. During the evaluation of our method, particularly in the last three experiments, we will investigate how this difference in diversity may influence the classification performance.

**Table 2:** Grouping of different heartbeat types according to the AAMI.

AAMI class	Heartbeat type	MIT-BIH	INCART	European ST-T
Non-ectopic beats (N)	Normal beat	$\times$	$\times$	$\times$
	Left bundle branch block beat	$\times$		
	Right bundle branch block beat	$\times$	$\times$	
	Atrial escape beat	$\times$		
	Nodal escape beat	$\times$	$\times$	
Supraventricular ectopic beats (S)	Atrial premature beat	$\times$	$\times$	
	Aberranted atrial premature beat	$\times$		$\times$
	Nodal premature beat	$\times$		$\times$
	Supraventricular premature	$\times$	$\times$	$\times$
Ventricular ectopic beats (V)	Premature ventricular contraction	$\times$	$\times$	$\times$
	Ventricular escape beat	$\times$		
Fusion beats (F)	Fusion of ventricular and normal beat	$\times$	$\times$	$\times$
Unknown beats (Q)	Paced beat	$\times$		
	Fusion of paced and normal beat	$\times$		
	Unclassified beat	$\times$	$\times$	$\times$

#### Experiments 1 and 2

To evaluate the stability of our approach, we conduct these two experiments on the two distinct databases: MIT-BIH and INCART.

The distribution of ECG records from which the studied heartbeats are extracted is outlined in Table 3. This table encompasses a total of 75 ECG records from all 32 patients, with 46 records allocated for training and 29 records for testing. It’s worth noting that the number of records exceeds the number of patients, as one or more records are obtained from a single patient. This distribution ensures that two records of a particular patient are only present in the same subset. Similarly, for the MIT-BIH database, there are 43 ECG records distributed as 22 records for training and 21 records for testing. The records (102, 104, 107, and 217) that contain paced beats, as well as record 108, are not included in our experiments.

After removing the unknown beats annotated as (Q), and considering the very low number of beats classified as class (F), representing less than $0.4\%$ of the total number of heartbeats extracted from all databases, we decided to remove these heartbeats as well. The remaining heartbeats, categorized into 11 types, are grouped into three main classes: Non-ectopic beats (N), Supraventricular ectopic beats (S), and Ventricular ectopic beats (V). These heartbeats are distributed as follows:

- MIT-BIH database: 85,134 (N), 2432 (S), and 6029 (V), totaling 93,895 heartbeats.

- INCART database: 153,422 (N), 1958 (S), and 19,990 (V), totaling 175,370 heartbeats.

- From the European ST-T database: 47,000 (N), 653 (S), and 1103 (V), totaling 48,756 heartbeats.

We note that from the MLII 47 records of the European ST-T database, we extracted all Supraventricular and Ventricular ectopic beats. Due to the length of these records (each lasting 2 h), we randomly selected 500 Non-ectopic (N) beats per record.

**Table 3:** Sizes of the training and testing subsets of all extracted heartbeats form MIT-BIH, INCART, and European ST-T databases.

Database	Subset	ECG records	Number of heartbeats
MIT-BIH	Training	101, 106, 109, 112, 114, 115, 116, 118, 119, 122, 124, 201, 203, 205, 207, 208, 215, 220, 222, 223, 230, 232	51066
	Testing	100, 103, 105, 111, 113, 117, 121, 123, 200, 202,209, 210, 212, 213, 214, 219, 221, 228, 231, 233, 234	42529
INCART	Training	I03, I04, I05, I08, I09, I10, I11, I12, I13, I14, I16, I17, I18, I19, I25, I26, I27, I28, I33, I34, I35, I36, I37, I44, I45, I46, I47, I48, I54, I55, I56, I68, I69, I70, I71, I72, I73, I74, I75	104804
	Testing	I01, I02, I06, I07, I15, I20, I21, I22, I23, I24, I29, I30, I31, I32, I38, I39, I40, I41, I42, I43, I49, I50, I51, I52, I53, I57, I58, I59, I60, I61, I62, I63, I64, I65, I66, I67	70566
European ST-T	Testing	e0103, e0104, e0105, e0106, e0108, e0110, e0111, e0112, e0113, e0114, e0115, e0116, e0118, e0119, e0121, e0122, e0123, e0124, e0125, e0126, e0127, e0129, e0133, e0136, e0139, e0147, e0148, e0151, e0154, e0155, e0159, e0161, e0162, e0163, e0166, e0170, e0601, e0602, e0604, e0605, e0606, e0609, e0610, e0611, e0612, e0613, e0615	48756

Table 4 presents the precision, sensitivity, and F1-score of the heartbeat classification within the testing subsets of the four experiments described previously. For the MIT-BIH database, the classification achieved a high accuracy of 99.09%, with an average precision of 94.25%, average sensitivity of 90.27%, and an average F1-score of 92.13%. Similarly, for the classification of INCART heartbeats, a high accuracy of 98.37% was attained, along with an average precision of 81.72%, average sensitivity of 77.24%, and an average F1-score of 79.37%.

In Exp.1 (MIT-BIH), the model demonstrated strong classification performance, achieving a macro average specificity of 97.47%. Among the classes, the highest specificity was observed for class S (99.86%), followed by class V (99.73%) and class N (92.81%). In Exp.2 (INCART), the model maintained high overall performance, achieving a macro average specificity of 95.54%. Class S achieved a high specificity of 98.81%, while class V and class N obtained specificities of 96.20% and 91.62%, respectively.

The analysis of the AUC metrics for experiments (Exp.1) and (Exp.2), which represent weak validation on the MIT-BIH and INCART databases respectively, shows promising results. For class (N), both experiments achieved an AUC of 1, indicating perfect classification performance for normal beats. The supraventricular ectopic (S) beats also demonstrated strong AUC values (0.99 for experiment Exp.1 and 1 for experiment Exp.2), suggesting the model’s capability to accurately classify these less frequent beats. For the ventricular ectopic (V) beats, both experiments reached an AUC of 1, indicating flawless classification. Overall, these results highlight the robustness of our model when tested on the two databases with diverse heartbeats and conditions, supporting the effectiveness of our approach in classifying ECG signals.

**Figure 6:**
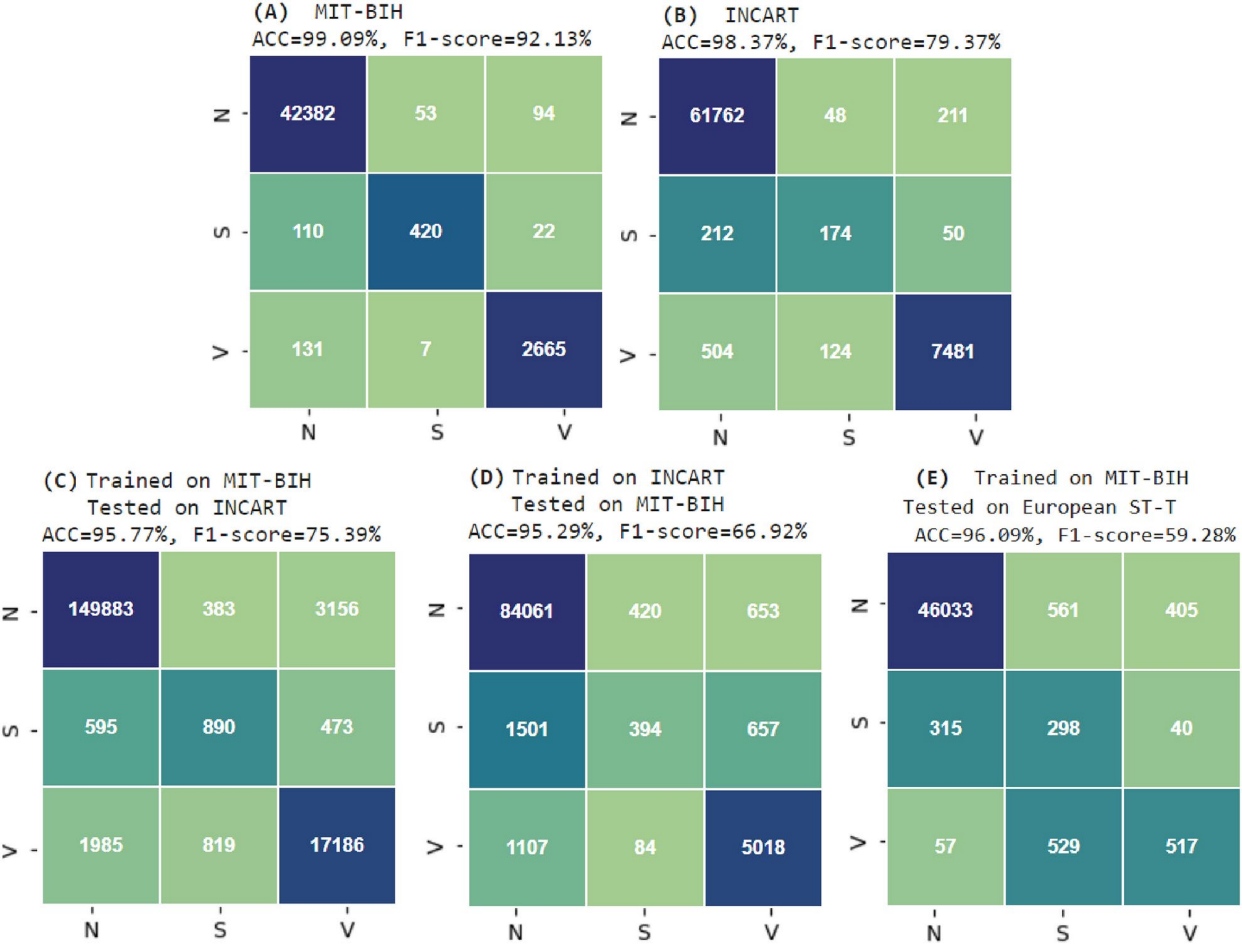
Confusion matrices: (**A**) and (**B**) Represent Weak Validation on MIT-BIH and INCART Databases, Respectively; (**C**), (**D**), and (**E**) represent strong validation on INCART, MIT-BIH, and European ST-T databases, respectively.

**Figure 7:**
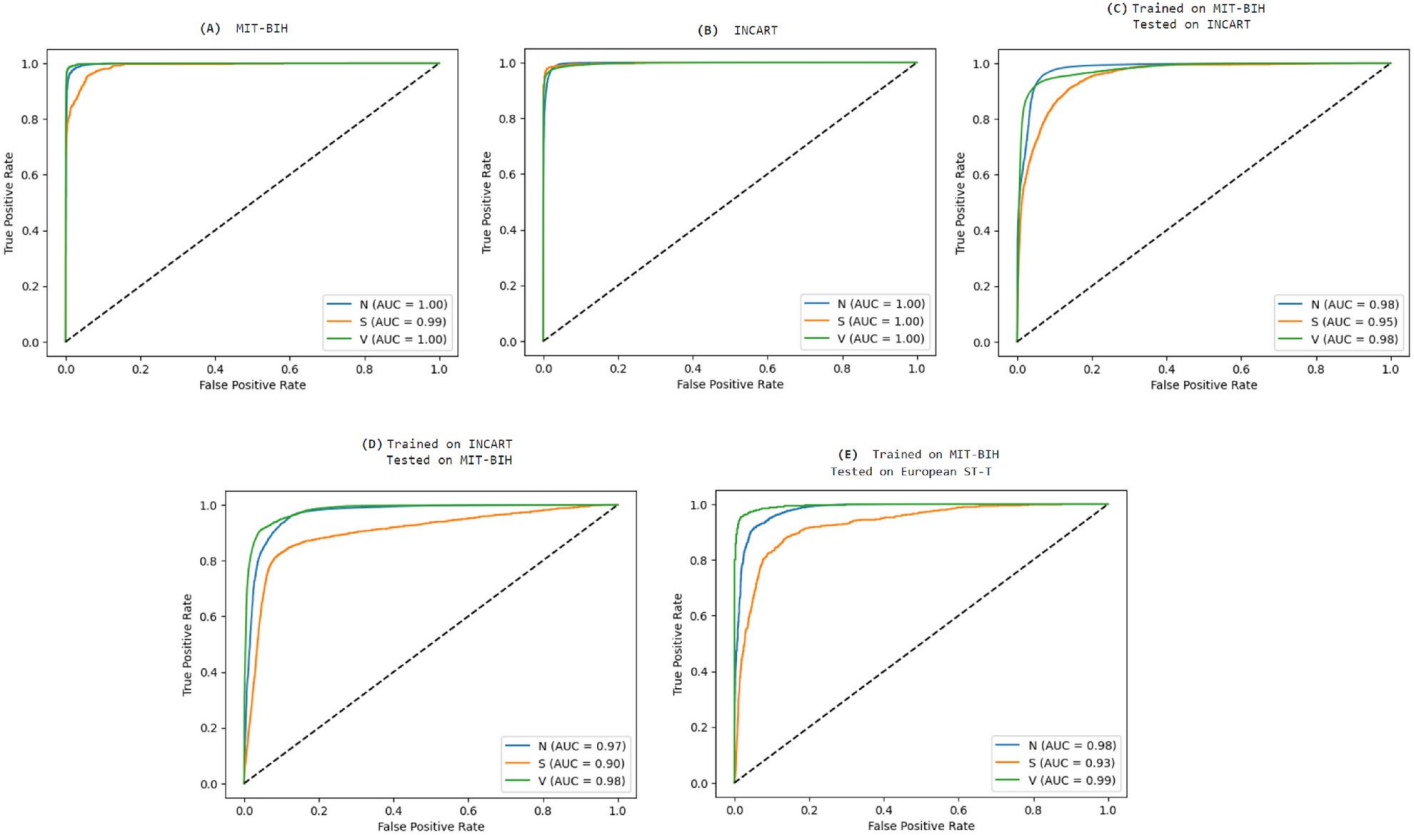
ROC curves and AUC metrics: (**A**) Weak validation on MIT-BIH, (**B**) Weak Validation on INCART, (**C**) Strong Validation Training on MIT-BIH and Testing on INCART, (**D**) Strong Validation Training on INCART and Testing on MIT-BIH, and (**E**) Strong Validation Training on MIT-BIH and Testing on ST-T.

The confusion matrices illustrating the final results of classification obtained in these two experiments are displayed in Subfigures (A) and (B) of Fig. 6. The receiver operating characteristic (ROC) curves are shown in Subfigures (A) and (B) of Fig. 7.

**Table 4:** Performance of the classification of randomly selected testing heartbeat subsets across the entirety of both MIT-BIH and INCART databases.

Experiment	Database	Class	Precision	Sensitivity	Specificity	F1-score	AUC	Accuracy
Exp.1	MIT-BIH	N	99.43%	99.65%	92.81%	99.54%	1.00	99.09%
		S	87.50%	76.09%	99.86%	81.40%	0.99	
		V	95.83%	95.08%	99.73%	95.45%	1.00	
		**Macro average**	**94.25%**	**90.27%**	**97.47%**	**92.13%**	**0.99**	
Exp. 2	INCART	N	98.85%	99.58%	91.62%	99.22%	1.00	98.37%
		S	50.29%	39.91%	98.81%	44.50%	1.00	
		V	96.63%	92.23%	96.20%	94.39%	1.00	
		Macro average	**81.72%**	**77.24%**	**95.54**%	**79.37%**	**1.00**	

Significant values are given in bold.

It is evident that the proposed method achieved better performance with the MIT-BIH database compared to the INCART. This discrepancy can be attributed to the fact that the heartbeats studied in the MIT-BIH dataset are extracted from 43 different patients, providing greater variability in terms of heartbeat morphology compared to those extracted from the INCART dataset, which contains only 32 patients. The increased diversity in the MIT-BIH dataset likely facilitates a more robust training process, enabling the model to generalize better to unseen data and ultimately resulting in higher classification performance.

#### Experiments 3, 4, and 5

Here we introduce our most challenging experiment. We have trained our model using all heartbeats sourced from either the MIT-BIH or INCART database, and subsequently assessed its performance using heartbeats from the alternate database. Additionally, we extended this validation by testing the model on the European ST-T database after training it on the MIT-BIH database. Nevertheless, the constrained patient pool in both databases, from which heartbeats are extracted, presents a challenge exacerbated by the diverse circumstances of ECG acquisition, variations in sampling rates, and disparities in recording equipment.

**Table 5:** Performance of the realistic classification of ECG heartbeats from the last experiment.

Train database	Test database	Class	Precision	Sensitivity	Specificity	F1-score	AUC	Accuracy
MIT-BIH	INCART	N	98.31%	97.69%	88.24%	98.00%	0.98	95.77%
		S	42.54%	45.45%	99.30%	43.95%	0.95	
		V	82.57%	85.97%	97.66%	84.23%	0.98	
		**Macro Avg**	**74.47%**	**76.37%**	**95.07%**	**75.39%**	**0.97**	
MIT-BIH	European ST-T	N	99.20%	97.94%	70.23%	98.57%	0.98	96.09%
		S	21.47%	45.64%	99.45%	29.20%	0.93	
		V	53.74%	46.87%	98.50%	50.07%	0.99	
		**Macro Avg**	**58.14%**	**63.48%**	**89.39%**	**59.28%**	**0.96**	
INCART	MIT-BIH	N	96.99%	98.74%	78.81%	97.86%	0.97	95.29%
		S	43.88%	15.44%	98.35%	22.84%	0.90	
		V	79.30%	80.82%	99.32%	80.08%	0.98	
		Macro Avg	**73.39%**	**65.00%**	**92.16%**	**66.92%**	**0.95**	

Significant values are given in bold.

Table 5 demonstrates the precision, sensitivity, F1-score, and accuracy achieved in our latest experiment, focusing on realistic heartbeat classification using our method. This experiment encompasses the full set of heartbeats categorized into three classes (N), (S), and (V) as defined by the AAMI association (see Table 2). Initially, we trained our model using all heartbeats of these classes extracted from the MIT-BIH database, then tested it on corresponding heartbeats from the INCART database. Subsequently, we repeated this process in reverse order. Notably, the highest performances were achieved when the model was trained on the MIT-BIH database and tested on the INCART database, resulting in an accuracy rate of 95.77% and an F1-score of 75.39%. Conversely, when the process was reversed-training the model on the INCART database and testing it on the MIT-BIH database-lower performances were observed, with an accuracy rate of 95.29% and an F1-score of 66.92

Additionally, when the model was trained on the MIT-BIH database and tested on the European ST-T database, we obtained an accuracy rate of 96.09%. Class N achieved the highest precision at 99.20% and an F1-score of 98.57%, reflecting the model’s strong performance in identifying normal beats. However, challenges were observed in detecting abnormal classes S and V. Class S had a low precision of 21.47% and an F1-score of 29.20%, while class V achieved slightly better results with a precision of 53.74% and an F1-score of 50.07%. Nonetheless, the high specificity values for the classes S and V at 97.15% and 97.18% , respectively.

In terms of specificity, when trained on MIT-BIH and tested on INCART, the model achieved an encouraging macro average specificity of 95.07%, effectively differentiating all classes, particularly class S and V with 99.30% and 97.66%, respectively. Similarly, when tested on the European ST-T database, the macro average specificity was 89.39%, with the method still maintaining high specificity for classes S and V at 99.45% and 98.50%, respectively. Even in the case of training on INCART and testing on MIT-BIH, the model achieved a 92.16% macro average specificity, with class V showing high performance at 99.32%. These results highlight the model’s robustness in reducing false positives in these two classes. However, class N showed a slightly lower specificity, such as 78.81% in the INCART-to-MIT-BIH experiment, potentially due to differences in heartbeat variability across the datasets.

The illustrated AUC and the receiver operating characteristic (ROC) curve shown in Fig. 7 for the performed cross-validation experiments demonstrate the robustness of the model, achieving encouraging results for the all classes, with values ranging from 0.90 to 0.99. These results highlight the model’s ability to generalize effectively across diverse datasets, even when faced with challenges such as limited patient numbers, differences in sampling rates, and imbalanced class distributions. This reinforces the potential of the proposed method for accurate heartbeat classification in a variety of clinical scenarios.

It is worth mentioning that our model consistently maintained an accuracy exceeding 95% in all experiments. However, the superior performance observed when training on the MIT-BIH database can be attributed to the diversity of heartbeat types (see Table 2). Despite the training subset extracted from the MIT-BIH database (93895) being smaller than the testing subset from the INCART database (75370), the model performed exceptionally well. The model’s performance was somewhat lower when tested on the European ST-T database, which could be due to the lower sampling rate of the ECG signals in the European ST-T database. Sub-figures (C), (D), and (E) in Fig. 6 show the confusion matrices of the three latest experiments corresponding to the results presented in Table 5.

### Impact of the PMAT algorithm on ECG classification performance

In order to understand the efficacy of the PMAT algorithm in ECG heartbeat classification, two extra experiments were done on MIT-BIH database training and testing subsets shown in Table 3. In the first experiment (**Exp. A**), the PMAT algorithm together with CNN architecture was used, but without handcrafted features. The second experiment (**Exp. B**) made use of handcrafted features only and did not use the PMAT algorithm and the CNN architecture. These experiments were aimed at demonstrating how PMAT and handcrafted features could be used effectively in enhancing the accuracy of classification while on their own. A detailed comparison of the results obtained from these experiments is presented in Table 6.

It’s clear that the Exp. A achieve an overall higher accuracy and shows improved performance for the (V) class. The Exp. B achieves better performance for (S) class. This superior performance for (S) class in Exp. B is expected since clinical analysis of this type of heartbeat strongly relies on temporal features such as the Pre-RR, Post-RR intervals ...ect. which play an important role in differentiating supraventricular ectopic beats.

N and S heartbeats share certain morphological similarities, especially in the QRS complex. However, they differ significantly in the regularity of the RR intervals preceding and following their R peaks, as well as in terms of the timing and presence of the P wave. The morphology of both can be captured from the ECG by PMAT. On the other hand, the timing features, such as irregular RR intervals, are handled by handcrafted features. This combined approach allows an effective distinction between the two classes for classification. In the case of the ventricular ectopic beats (V), the performance in Exp. A was better and also expected. This is because the identification of V heartbeats put more emphasis on the morphology of the heartbeat which can be captured in the images transformed by PMAT.

Since each method performs well for a different class, combining them logically leads to achieving better results across all three classes, as demonstrated by all the previous experiments.

**Table 6:** Comparison between Experiment A (PMAT + CNN) and Experiment B (Handcrafted Features + ANN) across all classes of MIT-BIH databse.

Class	Metric	Exp. A (PMAT + CNN)	Exp. B (handcrafted features)	Better approach
N	Precision	98.09%	**98.79%**	Exp. B
	Recall	**98.14%**	98.03%	Exp. A
	F1-Score	98.11%	**98.41%**	Exp. B
S	Precision	21.93%	**37.16%**	Exp. B
	Recall	16.85%	**37.50%**	Exp. B
	F1-Score	19.06%	**37.33%**	Exp. B
V	Precision	**83.48%**	73.54%	Exp. A
	Recall	**86.69%**	81.98%	Exp. A
	F1-Score	**85.05%**	77.53%	Exp. A
Overall	Accuracy	**96.46%**	96.32%	Exp. A
	Macro average F1-Score	67.41%	**71.09%**	Exp. B
	Weighted avg F1-Score	96.36%	**96.40%**	Almostly similar

Significant values are given in bold.

## Visual explanation

**Figure 8:**
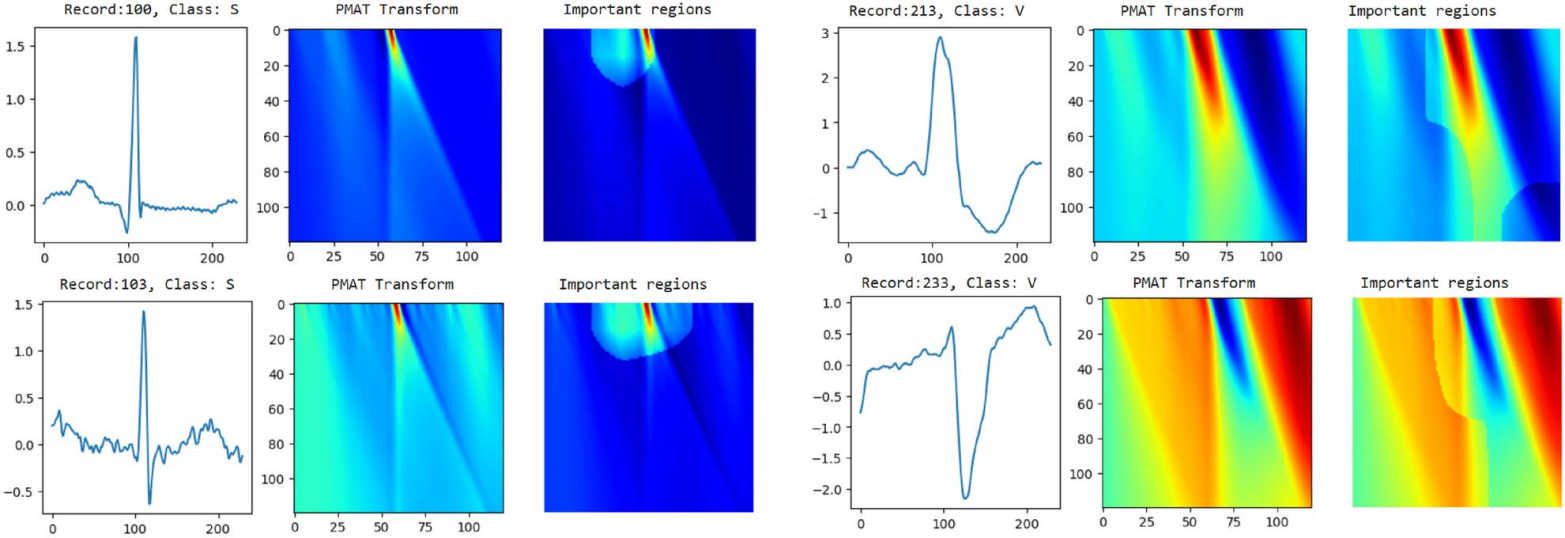
Grad cam.

This section uses Grad-CAM (Gradient-weighted Class Activation Mapping)^21^ to visualize and locate the most important regions in the transformed images of classified heartbeats. Using the gradients of the predicted class flowing into the final convolutional layer of the trained model, Grad-CAM generates a heat map that highlights the specific areas of the image that are the most responsible for the decision of the model. Four heartbeats were analyzed (see Fig. 8): two heartbeats of class S and two of class V, extracted, respectively, from records 100, 103, 213, and 233. For the first S heartbeat, the model focuses on the region preceding the R peak and the R peak itself. This is likely due to the overlap of the P wave with the T wave of the preceding heartbeat. In the second S heartbeat, the model focuses on a narrow area around the R peak, even in the absence of the P wave in that area. In both cases, whether the overlap of the P wave with the T wave in the first case or its absence in the second, this characteristic is considered a distinguishing feature of class S.

In the last two cases of heartbeats of class V, the model focuses on a broader region surrounding the R peak. This is likely due to the larger QRS complex, a key feature of ventricular ectopic beats. Additionally, the proximity of the P wave to the R peak may also contribute to the model’s focus in these areas.

### Comparison with other existing methods

Table 7 provides a summary of a comparison between the classification results obtained by our method on both the MIT-BIH and INCART databases and those obtained by other methods. While many of these methods have demonstrated good performance, they have often been applied primarily to the MIT-BIH database, making it challenging to verify their stability across different databases. Two methods presented in Huang et al.^3^ and Yoon et al.^10^ offer viable comparisons with our method as they utilize STFT and CWT transforms, respectively, which are similar to our PMAT transform. However, the first method^3^, employing STFT, was tested on a very limited subset of size 2520 and not on the entire MIT-BIH database, thus resulting in comparisons that diverge from our results. On the other hand, the second method^10^, utilizing the CWT transform, was applied to a larger database and yielded promising results. Nonetheless, the authors did not assess the method’s stability across different databases, raising questions about its generalizability. From Table 7, our method achieves an accuracy of 99.09% and an F1-score of 92.13% on the MIT-BIH database, and an accuracy of 98.37% with an F1-score of 79.37% on the INCART database. Compared to other recent works, it is evident that our approach, utilizing the proposed PMAT and a 2D-CNN model, has yielded encouraging results.

Fan et al.^22^ reported results that outperform our proposal. However, their evaluation was performed on a single dataset, MIT-BIH. In contrast, our method has been tested across multiple datasets, which offers a more comprehensive of its robustness and generalizability. Unfortunately, the authors of^22^ did not provide evaluations on additional databases, which limits the ability to give a full comparison.

**Table 7:** Comparison of the results obtained on MIT-BIH and INCART databases with other works.

Method	Year	Database	Used techniques	Accuracy	F1-score
Chazal et al.^8^	2004	MIT-BIH	Linear discriminants based model	86.20%	–
Qibin Z. and Liqing Z.^9^	2005	MIT-BIH	SVM + Wavelet Transform	99.68%	–
Melgani et al.^23^	2008	MIT-BIH	SVM + PSO	91.67%	–
Übeyli et al.^24^	2009	MIT-BIH	RNN	98.06%	–
Dutta et al.^25^	2010	MIT-BIH	CC-based feature extraction + SVM	96.12%	–
Yang et al.^26^	2018	MIT-BIH	PCANet + SVM	97.77%	88.20%
Huang et al.^3^	2019	MIT-BIH	STFT + 2D-CNN model	99.00%	–
Van et al.^27^	2020	MIT-BIH	Novel Parallel Neural Network	97.70%	86.40%
Fan et al.^22^	2022	MIT-BIH	MBLS-based model	99.12%	93.19%
Yoon et al.^10^	2023	12-lead ECG Database^28^	CWT + Bimodal 2D-CNN	95.74%	95.20%
This work	2023	MIT-BIH	Proposed PMAT + 2D-CNN model	**99.09**%	**92.13**%
This work	2023	INCART	Proposed PMAT + 2D-CNN model	**98.37**%	**79.37**%

Significant values are given in bold.

## Conclusion

Within our paper, we introduce the Progressive Moving Average Transform (PMAT) as a groundbreaking method designed to convert 1D discrete signals into 2D representations, or images. PMAT stands out due to its flexibility, offering three distinct variants: Left PMAT, Right PMAT, and Centered PMAT. These variants provide a versatile alternative to traditional transforms like Continuous Wavelet Transform (CWT) and Short Time Fourier Transform (STFT), offering researchers and practitioners a range of options for feature extraction and signal analysis.

To assess the effectiveness of PMAT, we integrated it into a sophisticated framework based on 2D Convolutional Neural Networks (2D-CNN). Our primary objective was to employ this framework for the classification of heartbeats obtained from two prominent databases: MIT-BIH and INCART. By utilizing PMAT within the 2D-CNN architecture, we aimed to leverage its unique properties for robust feature extraction, thereby enhancing the classification accuracy of our model.

Through rigorous experimentation and analysis, we obtained competitive results, underscoring the efficacy of PMAT in extracting discriminative features from the input signals. Our findings demonstrate that PMAT-equipped 2D-CNN models achieve commendable performance in classifying heartbeats from both the MIT-BIH and INCART databases.

In addition to evaluating PMAT’s performance in isolation, we conducted a comparative study with established transforms such as CWT and STFT. This comparative analysis provided valuable insights into the relative strengths and weaknesses of each method. Notably, our results indicate that PMAT performs on par with CWT while exhibiting a slight advantage over STFT in terms of classification accuracy and feature extraction capabilities.

Overall, our research highlights the promising potential of PMAT as a versatile tool for signal processing and classification tasks, particularly in the domain of biomedical signal analysis. The integration of PMAT into 2D-CNN frameworks opens up exciting avenues for further exploration and applications in various fields requiring robust and efficient signal processing techniques.

Future research should focus on optimizing PMAT parameters for various signal types, conducting comparative studies against established transforms, exploring applications in tasks such as denoising and classification, extending to multimodal data, implementing real-time solutions, assessing robustness, and exploring novel applications in fields like biomedical signal analysis and environmental monitoring. However, the implementation of a real-time tool requires an efficient R peak detection algorithm, as most existing solutions, including ours, rely on R peaks that are manually annotated by experts and provided within the studied databases. Although several algorithms exist for this task, we have not yet evaluated them across different databases. As part of our future work, we plan to study existing R peak detectors or explore the possibility of implementing a new one.

## Data Availability

The datasets and the source code are publicly available from the links: MIT-BIH database: https://www.physionet.org/content/mitdb/1.0.0/. INCART database: https://physionet.org/content/incartdb/1.0.0/. European ST-T Database: https://physionet.org/content/edb/1.0.0/. Source code: https://doi.org/10.5281/zenodo.14451283.
